# MicroRNA Profiling During Neural Differentiation of Induced Pluripotent Stem Cells

**DOI:** 10.3390/ijms20153651

**Published:** 2019-07-26

**Authors:** Katarzyna Kulcenty, Joanna P Wroblewska, Marcin Rucinski, Emilia Kozlowska, Karol Jopek, Wiktoria M Suchorska

**Affiliations:** 1 Radiobiology Lab, Department of Medical Physics, Greater Poland Cancer, 61-866 Poznań, Poland; 2 Department of Pathology, Poznan University of Medical Sciences and Greater Poland Cancer Center, Garbary 15th Street, 61-866 Poznan, Poland; 3 Department of Histology and Embryology, Poznan University of Medical Sciences, Swiecickiego 6 Street, 60-781 Poznan, Poland; 4 Department of Molecular Biomedicine, Institute of Bioorganic Chemistry, Polish Academy of Sciences, Noskowskiego 12/14 Str., 61-704 Poznan, Poland; 5 Department of Electroradiology, Poznan University of Medical Sciences, Garbary 15th Street, 61-866 Poznan, Poland

**Keywords:** human induced pluripotent stem cells, neural differentiation, neural stem cells, microRNA

## Abstract

MicroRNAs (miRNA) play an essential role in the regulation of gene expression and influence signaling networks responsible for several cellular processes like differentiation of pluripotent stem cells. Despite several studies on the neurogenesis process, no global analysis of microRNA expression during differentiation of induced pluripotent stem cells (iPSC) to neuronal stem cells (NSC) has been done. Therefore, we compared the profile of microRNA expression in iPSC lines and in NSC lines derived from them, using microarray-based analysis. Two different protocols for NSC formation were used: Direct and two-step via neural rosette formation. We confirmed the new associations of previously described miRNAs in regulation of NSC differentiation from iPSC. We discovered upregulation of miR-10 family, miR-30 family and miR-9 family and downregulation of miR-302 and miR-515 family expression. Moreover, we showed that miR-10 family play a crucial role in the negative regulation of genes expression belonging to signaling pathways involved in neural differentiation: WNT signaling pathway, focal adhesion, and signaling pathways regulating pluripotency of stem cells.

## 1. Introduction

Stem cells, due to their pluripotent character and nearly unlimited potential to differentiate, are a useful tool in basic research, disease modeling, drug toxicity tests, and regenerative medicine. A turning point in stem cell research occurred with two seminal studies, which demonstrated that overexpression of four transcription factors (OSKM: *OCT3/4*, *SOX2*, *KLF4* and *c-MYC*) induces pluripotency in adult murine [1] and human fibroblasts [2]. The resulting induced pluripotent stem cells (iPSC) have similar morphology, gene expression profile, and level of DNA methylation as embryonic stem (ES) cells. The most important shared trait of ESC and iPSC is their potential to differentiate into every cell type emerging from three germ layers. The fact that human somatic cells can be reprogrammed into pluripotent stem cells has dramatically changed the course of the development of stem cell therapy. The use of induced pluripotent stem cells obviates the two critical issues of ESC cells: Graft rejection and ethical controversies. Thus, shortly iPSC could be used both in regenerative medicine [3,4,5,6] and therapies based on gene replacement or gene correction [7,8,9,10]. Meanwhile iPSC has been widely used in modeling and pathogenesis studies of multiple diseases, especially problematic ones such as neurodegenerative diseases [11,12,13,14]. Differentiation of human induced pluripotent cells into neuronal cells is a potential source of neurons, astrocytes, and oligodendrocytes that can be used in regenerative medicine, to study the effects of drugs on the patient’s cells or to study the pathological mechanisms of diseases. 

The nervous system is a complex network of nerves and cells that transmits messages to and from the brain and spinal cord to different parts of the body. It originates during embryogenesis from a small number of neural stem cells (NSCc) [15]. The diversity of neural cell types is regulated mainly by transcription factors. Moreover, the amplitude of microRNAs (miRNA) in the human brain suggests that they may serve as a post-transcriptional regulator of gene expression [16]. It has been proven that specific miRNAs are involved in sustaining the pluripotent cell fate during early embryogenesis in mammals [17]. Their roles have also been proposed for other miRNAs in tissue-specific or organ-specific development [18]. MiRNAs are an abundant class of small non-coding RNA molecules, 18 to 25 nucleotides in length, that regulate expression at the post-transcriptional level by promoting mRNA degradation or blocking its translation into a functional protein. It has been suggested that miRNAs play a central role in controlling the stem cells and their differentiation by regulating the expression of stem cell regulators [19]. Studies on the molecular mechanisms underlying the neurogenesis can help to unravel the gene pathways involved in the differentiation process. 

In this context, we conducted differentiation of human induced pluripotent stem cells into neural stem cells. Then we carried out global miRNA expression analysis using the Affymetrix platform. We profiled the miRNAs which are crucial regulators of neural differentiation. We not only confirmed the involvement of previously described miRNAs characteristic for transition from ES to NSC: mir-302 family, hsa-miR-125b, hsa-miR-9, let-7, but we also pointed out others, which may be characteristic for iPSC to NSC transition: hsa-miR-512, hsa-miR-516b, hsa-miR-517a,b, hsa-miR-518e, hsa-miR-519c, miR-10 family (hsa-miR-125b-5p, hsa-miR-99a-5p, hsa-miR-100-5p), miR-30 family (hsa-miR-30a-5p, hsa-miR-30a-3p, hsa-miR-30c-2-3p), hsa-miR-217 and hsa-miR-219-3p. We also confirmed the involvement of those miRNAs in regulating WNT signaling pathway, TGF-β signaling pathway and focal adhesion during neural differentiation.

## 2. Results

### 2.1. Derivation of NSC from Human iPSC

**Figure 1:**
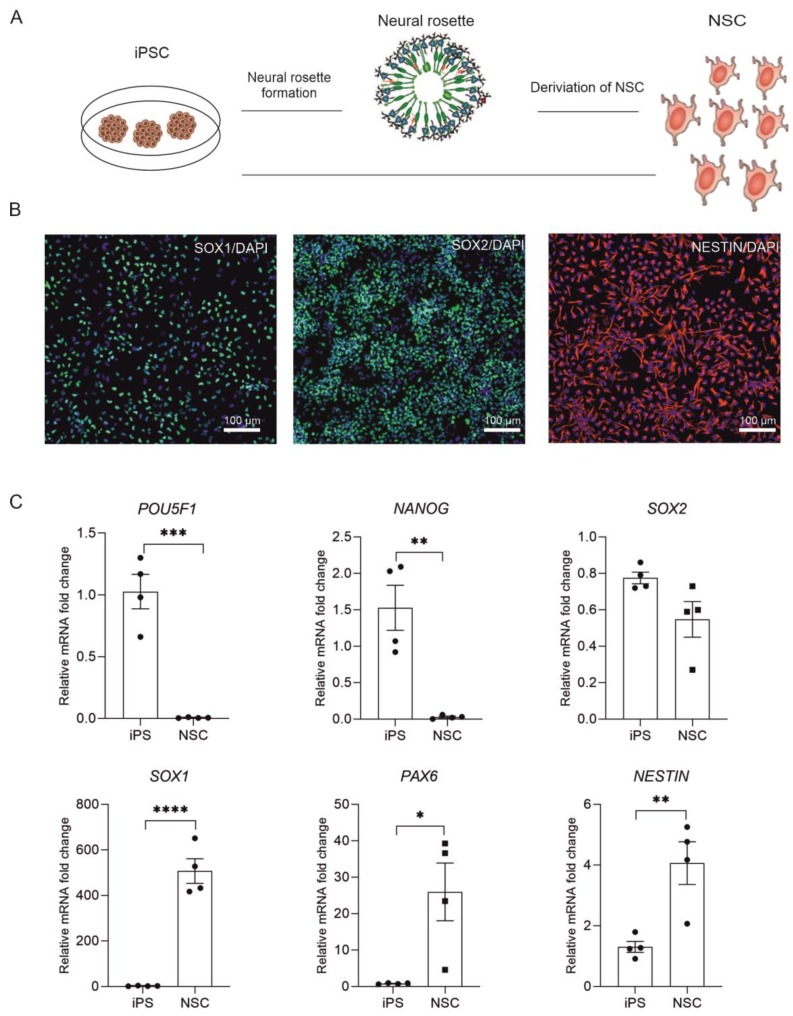
Human induced pluripotent stem cell lines were differentiated into neural stem cells via direct or indirect (neural rosette formation) protocol (**A**). Immunofluorescence staining for NSC markers confirmed successful differentiation (**B**). RT-qPCR analysis confirmed decreased expression of pluripotency markers (*POU5F1, NANOG*, *SOX2*) and increased NSC markers (*SOX1*, *PAX6*, *NESTIN*) in differentiated cells. Graphs represents mean ± SEM. Each dot represents an individual cell line (mean from 3 experiments conducted on 1 cell line) (**C**). Statistical analysis was conducted using t-student test (* *p* < 0.05, ** *p* < 0.01, *** *p* < 0.001, **** *p* < 0.0001).

For the derivation of NSC, we used one human induced pluripotent stem (iPSC) cell line, GPCCi001-A generated from primary human dermal fibroblasts according to previously established protocol [20] and one commercially available iPSC cell line—iPSC cell line ND41658*H (Coriell Cell Repository, NY, USA). IPSC were subjected to direct and indirect differentiation into neural rosettes and then NSC (Figure 1A).

During 7 days of culturing iPSC in differentiation, we observed specific changes in cell morphology. Between days 5 to 7 we observed the formation of neural rosettes, with a specific concentric arrangement of the cells, suggesting correct progression of cell differentiation (Figure 1A). After a week of differentiation, cells were expanded and subjected to immunofluorescence staining and RT-qPCR analysis, which confirmed their neural progenitor status. Immunofluorescent staining showed increased, expression of *NESTIN* (an early marker of neuronal cells), *SOX1* and *SOX2* in neural progenitors (Figure 1B). The RT-qPCR analysis revealed that the neural stem cells retained a high level of expression of *SOX2* gene (Figure 1C), similarly to iPSC. At the same time, we observed significant gain of expression of *PAX6* and *SOX1* genes, which are characteristic of multipotent neural stem cells. These results confirm the successful differentiation of iPSC into neural stem cells. 

### 2.2. miRNA Expression Profiling during Neural Differentiation from Induced Pluripotent Stem Cells

**Figure 2:**
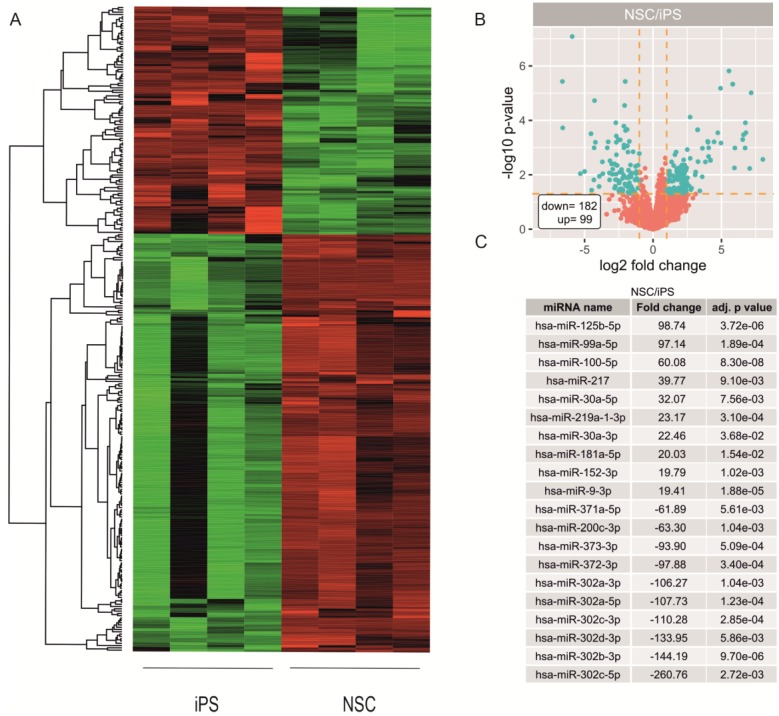
Microarray data analysis revealed a profile of miRNAs differentially expressed between iPSC and derived from them NSC cell lines (**A**). Volcano plot show the total miRNA expression profile of the iPSC and NSC cell lines. MiRNAs above the cut off are considered to be differentially expressed and are shown as blue dots (**B**). The table shows 20 miRNAs with the highest (10 miRNAs) and lowest (10 miRNAs) fold changes obtained from the differentially expressed miRNAs list (**C**).

To identify miRNAs that are involved in neural differentiation from pluripotent stem cells, a microarray assay using Applied Biosystems™ miRNA 4.1 Array Strips. The analysis included two different iPSC cell lines at different passages (obtained by authors serum-free GPCCi-001A [21] and ND41658*H (Coriell depository)) and NSC cell lines differentiated from them. The expression of miRNAs differs significantly between iPSC and NSC cells (Figure 2A). 

**Figure 3:**
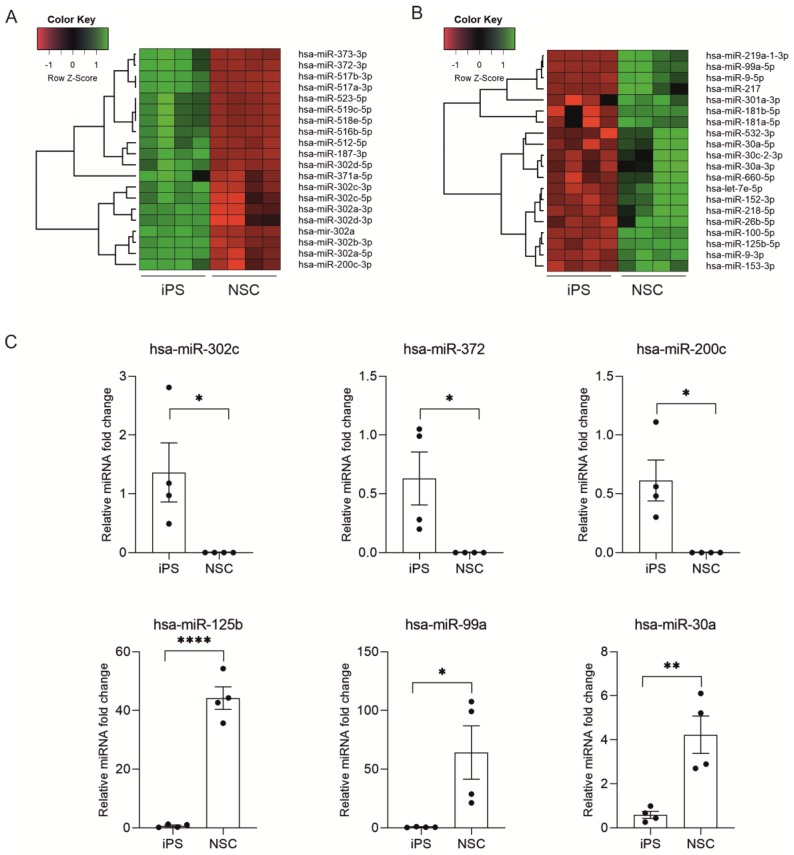
Real-time qPCR validation of microarray data. A) Microarray heat maps showing 20 highly downregulated (**A**) and upregulated (**B**) miRNAs in NSC cells. (**B**) Validation of 3 highly downregulated and 3 upregulated miRNAs in NSC by RT-qPCR. Graphs represents mean ± SEM. Each dot represents an individual cell line (mean from 3 experiments conducted on 1 cell line) (**C**). Statistical analysis was conducted using t-student test (* *p* < 0.05, ** *p* < 0.01, *** *p* < 0.001, **** *p* < 0.0001).

The whole miRNA expression profile is shown on Volcano plot (Figure 2B). The selected criteria were fold change >2 and *p*-value < 0.05. Based on these criteria we found 99 miRNA which were up-regulated and 182 miRNAs which were down-regulated in NSC versus iPSC. The top 20 of highly differentially expressed miRNAs are characterized by very high fold change values ranged for upregulated miRNAs from 19.89 to 98.78 and for downregulated from −61.89 to −260.76 (Figure 2C). While analyzing 20 top downregulated miRNAs in NSC cells we found those belonging to miR-302 family (hsa-miR-302c-5p, hsa-miR-302b-3p, hsa-miR-302d-3p, hsa-miR-302c-3p, hsa-miR-302a-3p, hsa-miR-302a, hsa-miR-302d-5p), miR-515 family (hsa-miR-519c-5p, hsa-miR-523-5p, hsa-miR-518e-5p) and hsa-miR-372-3p, hsa-miR-373-3p, hsa-miR-371-5p (Figure 3A). 

Among 20 top miRNAs which are highly upregulated in NSC cells we found those belonging to miR-10 family (hsa-miR-125b-5p, hsa-miR-99a-5p, hsa-miR-100-5p), miR-30 family (hsa-miR-30a-5p, hsa-miR-30a-3p, hsa-miR-30c-2-3p), miR-9 family (hsa-miR-9-3p, hsa-miR-9-5p), hsa-miR-217, hsa-miR-219-1-3p, hsa-let-7e-5p (Figure 3B). The accuracy of these results was confirmed by RT-qPCR analysis (Figure 3C). 

### 2.3. Biological Processes Regulated by miRNA During Neural Differentiation

**Figure 4:**
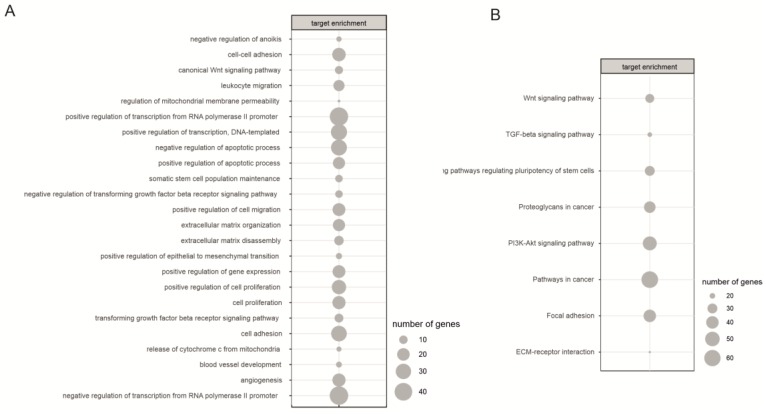
Analysis of biological processes regulated by differentially expressed miRNAs target genes (DAVID software) revealed processes characteristic for neural differentiation (**A**—Gene Ontology database, **B**—KEGG database).

Next, to verify which processes are regulated by significantly differentiated miRNAs, DAVID analysis was performed. Firstly, targets for miRNAs were selected based on SpidermiR package where target genes were predicted by a screening of five miRNA-target gene databases (DIANA, Miranda, PicTar, TargetScan, miRTAR, miRwalk). We found 15779 predicted and validated target genes for our 281 differentially expressed miRNA. The actual expression level of target genes was determined using data from the Gene Expression Omnibus (GEO) database (accession number: GSE55107). By the assumption that increasing miRNA expression leads to decreasing target gene expression and vice versa, for further analysis we select those target genes in which the fold change was inversely correlated with the fold change of miRNA. With this approach, only 1502 target genes were selected. MiRNA targets were then assigned to Gene Ontology (GO) (Figure 4A) and KEGG database (Figure 4B). 

During neural differentiation of iPSC we found the involvement of processes related to the regulation of transcription, cell proliferation and regulation of cell death (apoptosis, anoikis). Moreover, GO analysis also revealed, that differentially expressed miRNAs are involved in regulating the expression of genes involved in processes connected to extracellular matrix organization (ECM) and disassembly, cell adhesion and positive regulation of epithelial to mesenchymal transition. Similar processes: Focal adhesion and ECM-receptor interaction were also revealed in the KEGG database (Figure 4). 

**Figure 5:**
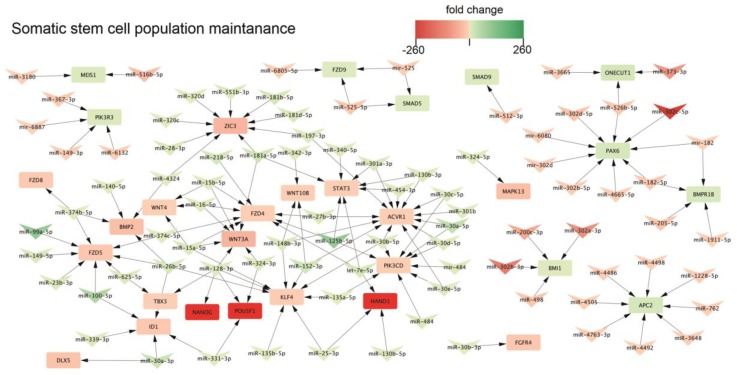
Signaling pathway regulating pluripotency of stem cells is modified during neural differentiation. Arrowheads represent differentially expressed miRNAs, and squares—negatively regulated genes.

The somatic stem cell population maintenance is one of the processes that changed during neural differentiation. Detailed analysis of this process revealed that expression of key genes of pluripotency: *NANOG*, *POU5F1* is decreased and inversely correlates with increased expression of hsa-miR-128-3p, hsa-miR-324-3p and hsa-miR-331-3p. On the other hand, an expression of neural marker *PAX6* is increased in NSC and inversely correlated by hsa-miR-302c-5p, hsa-mir-302d, hsa-miR-302d-5p, hsa-miR-302b-5p, hsa-miR-182-5p, hsa-miR-526-5p which are decreased in NSC (Figure 5). 

**Figure 6:**
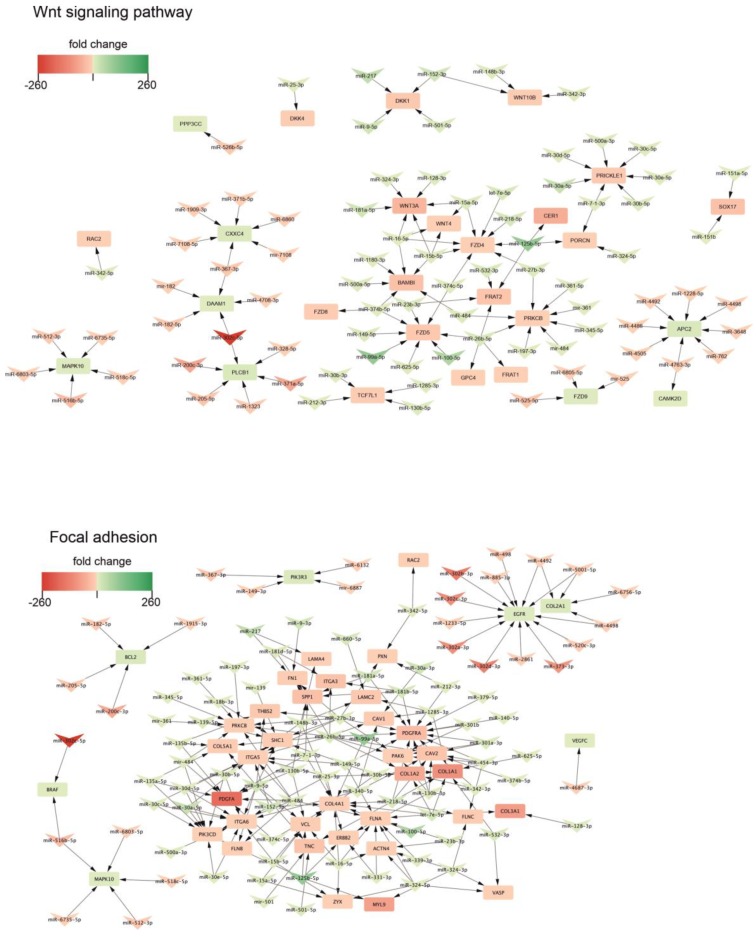
WNT signaling and focal adhesion are involved in the regulation of neural differentiation. Arrowheads represent differentially expressed miRNAs, and squares—negatively regulated genes.

In both databases an involvement of the WNT signaling pathway and TGF-beta signaling pathway was also confirmed. We found, that the WNT signaling pathway is regulated by miRNA belonging to highly enriched in NSC miR-10 family (hsa-miR-125b-5p, hsa-miR-99a-5p, hsa-miR-100-5p) (Figure 6 top). Many of miRNAs which are highly upregulated in NSC cells were cross-linked with genes belonging to the WNT signaling pathway thus displaying the negative regulation of this pathway. The overexpressed miRNAs negatively regulate the expression of genes like: *FZD4, FZD5, FZD8, BAMBI, WNT3A, WNT4, WNT10B*. Our finding also demonstrates the involvement of focal adhesion in neural differentiation (Figure 6 bottom). We found an extensive regulatory network between miRNAs which are highly expressed in NSC cells and their corresponding genes. Again, miR-10 family is the one mostly involved in the regulation of the pathway. Interestingly, we also found involvement of miR-302 family, which is highly downregulated during neural differentiation.

## 3. Discussion

The unlimited ability of iPSC to proliferate and the potential to differentiate into various cell types of all three germline lines give hope for their use in regenerative medicine or disease modeling. The primary purpose of using stem cells in regenerative medicine is to improve the functioning of a given tissue by replacing damaged cells with new cells and stimulating the endogenous repair process. Animal studies have shown that it is possible to replace degenerated nerve cells using stem cells, which in many cases leads to functional improvement. Many applications of neuronal stem cells are potential in the therapy of neurodegenerative diseases, including Parkinson’s disease, Alzheimer’s disease, Huntington’s disease, multiple sclerosis, and stroke patients. Thus, understanding the molecular mechanisms standing behind the neural differential process is an important issue. Many research has been conducted concerning the transcriptomic profiling of cells during neurogenesis and thus it has brought us closer to understanding the mechanism responsible for the transition from iPSC to NSC. However, the regulation of this transition on the miRNA level has not yet been fully understood. Here we report for the first time the global miRNA profiling of iPSC and their derivatives—neural progenitors. Similar high throughput experiments were also conducted, however concerning human mesenchymal stem cells [21] or adipose tissue-derived stem cells [22]

As for now most of the studies addressing the miRNA role in neurogenesis were conducted using animal models. Experiments on mouse embryos revealed a significant part of mmu-miR-9 and mmu-miR-124 in promoting neurogenesis and NSC differentiation [23,24,25,26]. The knowledge of human neural development was not accessible due to ethical issues concerning the research on human embryos. However, with the increasing accessibility of human neural cells from human pluripotent stem cells there is an opportunity to study the role of miRNA in neurogenesis. We took advantage of this model and reprogrammed human induced pluripotent stem cells (both obtained in our lab GPCCi001-A [20], and commercially available ND41658*H (Coriell Cell Repository, NY, USA) into neural progenitors (Figure 1). A miRNA profiling using microarray assay revealed decreased expression of miRNAs belonging to miR-302 family, miR-515 family and hsa-miR-372-3p, hsa-miR-373-3p, hsa-miR-371-5p. It has been previously shown, that miR-302 blocks neural induction mainly by contributing to BMP signaling [27]. Previous miRNA profiling of embryonic stem cells (ESC) from both human and mouse revealed that mir-302 cluster, and corresponding mouse mir-290–295, are the most abundant miRNA transcript in ESC [17]. Lipchina et al. analyzed the mir-302 targets, and found that hsa-miR-302 is a positive regulator of pluripotency and self-renewal and it modulates TGF-β and BMP signaling during neural induction. They found that hsa-miR-302 promotes BMP signaling which leads to decrease in neural induction efficiency [27]. Moreover miR-302/367 also repress pro-neural transcription factor NR2F2 [28]. Literature data also validate other miRNAs which expression in our analysis is highly enriched in iPSC compared to NSC, confirming their inhibitory role in NSC differentiation. Studies on human embryonic stem cell and their differentiation capabilities to NSC revealed, that mmu-miR-200 acts on the same pathway which hsa-miR-302 does, and represses neural induction of ESC cells. It targets the transcriptor factor ZEB—a negative regulator of BMP/TGFβ signaling [29]. Our results strongly confirm, that the mechanism of neural induction in ESC cells and iPSC involves transcriptional regulation of the same miRNAs: miR-302 family, hsa-miR-371, hsa-miR-200. 

Moreover, our data also pointed out another group of miRNA, which are believed to be highly involved in negatively regulating NSC differentiation. Expression of hsa-miR-512, hsa-miR-516b, hsa-miR-518e, hsa-miR-519c and hsa-miR-523 is significantly decreased in NSC cells in comparison to iPSC (Figure 3). In mouse embryonic stem cells (mESC) the miR-290 cluster contributes to about 60% of the complete miRNAs in mESCs making it the most abundant miRNA family in mESCs [30]. The miR-290 cluster is conserved among human, chimpanzee, rat, mouse, dog and cow but stays restricted to placental mammals. Sequence comparisons indicated that in human ESC the mouse homologue of miR-290–295 is similar to miR-371 cluster and the moderately similar miR-512 cluster [31,32]. Sempere et al. with northern blot analysis of profiling of adult organs specific miRNAs from mouse and human and later in vitro studies revealed a miRNAs which are characteristic for the neural differentiation. Among them they found let-7, miR-9, miR-125b, miR-218 [16]. Let-7 miRNA family is a potent inhibitor of pluripotency and a promoter of the neural lineage [33]. The miR-9 interacts with gene regulatory network and thus induce the expression of neural differentiation program. This function may be in part dependent on the cooperation influence on the ATP-dependent BAF chromatin remodeling complex [34]. Moreover, miR-9 has been shown to target genes belonging to Notch signaling cascade—a critical pathway regulating neuronal development and expansion [35,36]. Boissart investigation has been demonstrated that hsa-miR-125 promotes the neural conversion of human ESCs into SOX1-positive NPs. They found that it depends on SMAD4 repression, suggesting that hsa-miR-125 is involved in the BMP-mediated signaling transduction of neural lineage commitment from ESCs [37].

We have shown, that apart from being involved in TGFβ pathway [38,39], differentially expressed miRNAs are also involved in other pathways connected to neural differentiation: WNT signaling pathway and focal adhesion. During the differentiation process, WNTs glycoproteins are secreted and regulate the differentiation and migration of neural progenitor cells. Thus, WNT signaling pathway participates in the formation of neuronal circuits [40]. It has been shown that WNT signaling enhances self-renewal of mouse embryonic and neural stem/progenitor cells. Kim et al. showed that undifferentiated ESC indicates a deficient level of endogenous WNT pathway and that WNT/β-catenin signaling increased during the neuronal differentiation. It is also important to point out, that it’s activity increased during formation of neural precursors and decreased in later neuronal differentiation [41]. Moreover, it has been shown, that some of neuron-specific transcription factors are regulated by WNT/β-catenin signaling pathway [42].

Our results indicate, that differentially expressed between iPSC and NSC miRNAs, are involved in regulating the expression of genes involved in processes connected to extracellular matrix (ECM) organization and disassembly, cell adhesion, focal adhesion and positive regulation of epithelial to mesenchymal transition. The ECM play a pivotal role in regulating stem cell differentiation, migration and proliferation during embryonic development. The ECM is a mixture of matrix molecules: fibronectins, collagens, laminins and proteoglycans that assemble into fibrils or other complex macromolecular arrays. Cell adhesion to the ECM transmits extracellular signals to stem cells via integrin receptors. The laminin family is one of the most essential ECM components within the neural stem cell niche [43]. Ma et al. focused on the involvement of the ECM components in the generation and migration of neural progenitors and neurite outgrowth of neurons during the neural specification of ESC. They found that laminin served as a potent stimulator of neural differentiation of ESCs while other tested ECM components showed similar, but much less enhancement of these events [44]. 

Despite numerous studies describing the involvement of miRNAs in neurogenesis, until now no global profiling of miRNAs during differentiation of induced pluripotent stem cells to neural stem cells was performed. As the induced pluripotent stem cells serves a great promise in regenerative medicine and therapies based on gene replacement or gene correction, especially in the field of neurodegenerative diseases, we believe that results presented in this study address the lack of the data concerning the molecular mechanisms underlying the neurogenesis process.

## 4. Materials and Methods 

### 4.1. Neural Induction of iPSC

Differentiation of GPCCi001-A cell line (generated and characterized in our laboratory [21]) was performed with one-step monolayer culture protocol. iPSC were seeded onto a six-well culture plate coated with Matrigel (1:20 solution) with a confluence less than 20%. Differentiation was performed in Neural Induction Medium consisting of Neurobasal Medium (Gibco, Waltham, MA, USA) supplemented with 1× Neural^®^ Induction Supplement (Gibco, Waltham, MA, USA). The medium was changed every other day for the first four days of differentiation, then every day. Cells were cultured for 7 days at 37 °C in a 5% O2 and 5% CO2 mixture until characteristic morphology of neural rosettes appeared. Next, cells were harvested with StemPro^®^ Accutase^®^ Cell Dissociation Reagent (Gibco, Waltham, MA, USA) digestion, and cultured further in Neural Expansion Medium (consisting of 1:1 mixture of Neurobasal Medium: DMEM F-12 and 1× Neural^®^ Induction Supplement).

Differentiation of commercially available ND41658*H iPSC (Coriell Cell Repository, NY, USA) iPSC cell line was also performed with one step monolayer culture, however using a STEMdiff™ Neural Induction Medium supplemented with STEMdiff™ SMADi Neural Induction Supplement and 10 µM Y-27632 (Stemcell Technologies, Vancouver, Canada). Briefly, 2 × 10^6^ iPSC were seeded onto a six-well culture plate coated with 1:25 Geltrex™ solution (Thermofisher Scientific, Foster City, CA, USA) and cultured according to protocol. The medium was changed every day for 9 days. When cells reached confluence of 90% were harvested with 0,5 mM EDTA in PBS for 10 min, and cultured for another two passages in the same medium. After third passage cells were cultured in STEMdiff™ Neural Progenitor Basal Medium supplemented with 2× Supplement A and 0.1× Supplement B (Stemcell Technologies, Vancouver, Canada) on Geltrex™ coated plates (1:25 solution). The medium was changed every other day.

### 4.2. Total RNA Isolation and RT-qPCR

Total RNA was purified with TRI Reagent solution according to the manufacturer’s protocol. One microgram of RNA was reverse transcribed using iScript™ cDNA Synthesis Kit (BioRad) and diluted 10× before PCR reaction. PCRs were performed with FastStart Taq DNA Polymerase Kit (Roche) and ReadyMix™ Taq PCR Reaction Mix (Sigma Aldrich). 

Real-Time quantitative PCR reactions were performed on cDNA template (obtained after the reverse transcription reaction) using a LightCycler^®^ 480 Probes Master set (Roche) and Universal Probe Library in combination with a set of matching target-specific PCR primers, according to the manufacturer’s protocol. Statistical analysis of obtained results was conducted in GraphPad Prism 6 (GraphPad Software), using t-student test (* *p* < 0.05, ** *p* < 0.01, *** *p* < 0.001, **** *p* < 0.0001).

### 4.3. RT-qPCR for miRNA Expression

To validate differentially expressed miRNA selected in microarray expression profiling miRNA from iPSC and NSC cell lines was extracted with miRNeasy Kit and RNeasy MinElute prepared with TaqMan™ MiRNA Reverse Transcription Kit (Applied Biosystems, Foster City, CA, USA). The quantitative real-time PCR analysis was performed with TaqMan miRNA Assay specific to chosen miRNA and TaqMan™ Universal PCR Master Mix (Applied Biosystems, Foster City, CA, USA). All real-time based analysis was performed on a Cobas Z480 device with LightCycler 480 Software (Roche, Basel, Switzerland). Statistical analysis of obtained results was conducted in GraphPad Prism 6 (GraphPad Software, San Diego, CA, USA), using student t-test.

### 4.4. Immunocytochemistry

Cells were plated on GeltrexTM Thermofisher Scientific, Foster City, CA, the USA coated cover glasses and cultured for 48 h. Next, cells were fixed with 4% formaldehyde solution, permeabilized with 0.5% Tween followed by incubation with 1% bovine serum albumin blocking solution for 30 min at room temperature and overnight incubation at 4 °C in a solution of primary antibody in 1%BSA/PBS. After incubation, the cells were washed and incubated for 1h at 37 °C with appropriate secondary fluorescent-labeled antibody (Jackson Immunoresearch, West Grove, PA, USA) solution in 1%BSA/PBS. Nuclei were counterstained with SlowFade^®^ Diamond Antifade Mountant (Thermofisher Scientific, Foster City, CA, USA). Cells were visualized under fluorescent Leica S5 confocal microscope (Leica, Weltzar, Germany). All antibodies used are listed in Table 1.

### 4.5. Microarray 

The profiling of miRNA expression was carried out using the microarray approach with Applied Biosystems™ miRNA 4.1 Array Strip (ThermoFisher Scientific, Waltham, MA, USA). Each microarray was designed according to miRBase Release 20 database that contained complementary probes to detect: 2578 human mature miRNA, 1996 human snoRNA, CDBox RNA, H/ACA Box RNA and scaRNA, 2025 human pre-miRNA. The complete procedure for preparing miRNA for hybridization was performed using the FlashTagTM Biotin HSR RNA Labeling Kit (ThermoFisher Scientific, Waltham, MA, USA). 150 ng of previously isolated miRNA was subjected to poly(A) tailing and biotin ligation procedure, according to producer protocol. Biotin-labeled miRNA were hybridized to Applied Biosystems™ miRNA 4.1 Array Strip (20 h, 48 °C). Then, the microarrays were subjected to a washing and staining procedure, according to the technical protocol, using the Affymetrix GeneAtlas Fluidics Station (Affymetrix, Santa Clara, CA, USA). The array strips were scanned using an Imaging Station of GeneAtlas System (Thermo Fisher Scientific, MA, USA). The preliminary analysis of the scanned chips was carried out by means of Affymetrix GeneAtlas Operating Software (Affymetrix, Santa Clara, CA, USA). The quality of the gene expression data was verified using the quality control criteria established by the software. Obtained CEL files from scanned microarrays were imported into downstream data analysis using BioConductor with relevant BioConductor libraries from statistical R programing language. The Robust Multiarray Average (RMA) normalization algorithm implemented in the “Affy” library was used for normalization, background correction, and calculation of the expression values of all of the examined miRNA [22]. Biological annotation was obtained from pd.mirna 4.1 library where annotated data frame object was merged with normalized data set, leading to a complete miRNA data table. Differential expression and statistical assessment were determined by applying the linear model for microarray data implemented in the “limma” library [23]. The selection criteria of a significantly changed gene expression were based on fold difference higher than absolute 2 and p-value after false discovery rate (FDR) correction <0.05. The result of such a selection was presented as volcano plot, showing the total number of up- and down-regulated miRNAs. Differentially expressed miRNA were also subjected to a hierarchical clusterization algorithm and visualized as heat map. Similar analysis was performed in relation to top 20 miRNA with the highest and lowest fold change values. Raw data files were also deposited in the Gene Expression Omnibus (GEO) repository at the National Center for Biotechnology Information (http://www.ncbi.nlm.nih.gov/geo/) under the GEO accession number GEO: GSE134061.

### 4.6. miRNA-Target Gene Prediction

To identify potential target genes for differentially expressed miRNA, a SpidermiR package was applied. Differentially expressed miRNAs were used as a query for searching target genes in the following databases: For predicted targets—DIANA, Miranda, PicTar, TargetScan, and for experimentally validated targets—miRTAR, miRwalk [24]. To determine the actual expression value of selected target genes, we have used data deposited in the Gene Expression Omnibus (GEO) database (accession number: GSE55107) from analogous experiment [25]. Obtained fold change values for mRNA were assigned to target genes data table. For further analysis, we have selected only those target genes for which fold change was inversely correlated with the fold change value of appropriate miRNA (cut-off criteria: fold ± 2, adjusted *p* value (adj.p.val.) < 0.05). Such selected set of target genes were subjected to functional annotation and clusterization using the DAVID (Database for Annotation, Visualization, and Integrated Discovery) bioinformatics tool [26,27]. Gene symbols of differentially expressed genes were uploaded to DAVID by the “RDAVIDWebService” BioConductor library [28], where DEGs were assigned to relevant GO terms, with subsequent selection of significantly enriched GO terms from GO BP Direct and KEGG database. The *p*-values of selected GO terms were corrected using Benjamini-Hochberg correction described as adjusted *p*-values [29]. Relevant GO ontological groups with adjusted *p*-values below 0.05 and N per group >5 were visualized using bubble plot. Interactions between miRNA and target genes, in selected GO BP or KEGG terms, were visualized using Cytoscape 3.7.1 [30].

## 5. Conclusions

In this experiment based on microarray profiling of miRNAs from 2 iPSC cell lines and 2 derived from them NSC cell lines we have discovered that apart of well-described miRNA which regulate the NSC differentiation from ESC (which we also validated in our iPSC-NSC model of differentiation), a new, not described previously miRNA may have a major impact in neural differentiation. We found that the expression of hsa-miR-512, hsa-miR-516b, hsa-miR-517a,b, hsa-miR-518e and hsa-miR-519c and hsa-miR-523 is highly decreased in NSC cells compared to iPSC, and thus it is believed that these miRNAs are the negative regulators of neural differentiation form iPSC. Moreover, among potential positive regulators of neural differentiation (high expression in NSC) we found miR-10 family (hsa-miR-125b-5p, hsa-miR-99a-5p, hsa-miR-100-5p), miR-30 family (hsa-miR-30a-5p, hsa-miR-30a-3p, hsa-miR-30c-2-3p), hsa-miR-217, hsa-miR-219-1-3p. It is also important to point out, that miR-10 family pay a crucial role in negative regulation of genes expression belonging to signaling pathways involved in neural differentiation: WNT signaling pathway, focal adhesion, signaling pathways regulating pluripotency of stem cells. However, it is important to point out that the results presented in this study are based on microarray profiling, and further studies are crucial to verify the miRNAs impact on miRNA-dependent biological pathways driving neural differentiation process. 

## Figures and Tables

**Table 1:** Primary and secondary antibodies used in immunofluorescent staining.

Antibody	Dilution	Company Cat# and RRID
Rabbit anti-SOX1	1:200	Cell Signaling Cat#9606
Rabbit anti-SOX2	1:400	Cell Signaling Cat#3579
Mouse anti-Nestin	1:500	Stemcell Technologies Cat#60091
Donkey Anti-Rabbit Alexa Fluor^®^ 488	1:1000	Jackson ImmunoResearch Cat#711-546-152
Donkey Anti-Mouse Alexa Fluor^®^ 594	1:1000	Jackson ImmunoResearch Cat#715-586-151

## Abbreviations

ECM	Extracellular matrix
KLF4	Kruppel-like factor 4
WNT10B	Wingless-type MMTV integration site family, member 10B
BMP	Bone morphogenetic protein
c-Myc	MYC proto-oncogene
DAVID	Database for Annotation, Visualization and Integrated Discovery
EDTA	Ethylene diamine tetra acetic
ESC	Embryonic stem cells
FZD4/5/8	Frizzled4/5/8
GEO	Gene expression omnibus
GO	Gene ontology
GPCCi001-A	Human induced pluripotent stem cell line created in Greater Poland Cancer Centre
iPSC	Induced pluripotent stem cells
KEGG	Kyoto Encyclopedia of Genes and Genomes
NANOG	Nanog homeobox
NR2F2	Nuclear receptor subfamily 2, group F, member 2
NSC	Neuronal stem cells
OCT3/4	Octamer-binding transcription factor 4
PAX6	Paired box protein 6
PBS	Phosphate buffer saline
PCR	Polymerase chain reaction
POU5F1	POU class 5 homeobox 1
RT-qPCR	Real time quantitative polymerase chain reaction
SMAD4	SMAD family member 4
SOX1	SRY (Sex determining region Y)-box 1
SOX2	SRY (Sex determining region Y)-box 2
TGF-β	Transforming growth factor beta
WNT	Wingless-type MMTV integration site family
WNT3A	Wingless-type MMTV integration site family, member 3A
WNT4	Wingless-type MMTV integration site family, member 4
ZEB	Zinc finger E-box binding homeobox
